# Potential for Mycorrhizae-Assisted Phytoremediation of Phosphorus for Improved Water Quality

**DOI:** 10.3390/ijerph18010007

**Published:** 2020-12-22

**Authors:** Jessica A. Rubin, Josef H. Görres

**Affiliations:** Plant and Soil Science, University of Vermont, Burlington, VT 05405, USA; jgorres@uvm.edu

**Keywords:** mycorrhizae, phosphorus, water quality, mycoremediation, phytoremediation, ecological restoration, ecological reconciliation, myco-phytoremediation, symbiosis

## Abstract

During this 6th Great Extinction, freshwater quality is imperiled by upland terrestrial practices. Phosphorus, a macronutrient critical for life, can be a concerning contaminant when excessively present in waterways due to its stimulation of algal and cyanobacterial blooms, with consequences for ecosystem functioning, water use, and human and animal health. Landscape patterns from residential, industrial and agricultural practices release phosphorus at alarming rates and concentrations threaten watershed communities. In an effort to reconcile the anthropogenic effects of phosphorus pollution, several strategies are available to land managers. These include source reduction, contamination event prevention and interception. A total of 80% of terrestrial plants host mycorrhizae which facilitate increased phosphorus uptake and thus removal from soil and water. This symbiotic relationship between fungi and plants facilitates a several-fold increase in phosphorus uptake. It is surprising how little this relationship has been encouraged to mitigate phosphorus for water quality improvement. This paper explores how facilitating this symbiosis in different landscape and land-use contexts can help reduce the application of fertility amendments, prevent non-point source leaching and erosion, and intercept remineralized phosphorus before it enters surface water ecosystems. This literature survey offers promising insights into how mycorrhizae can aid ecological restoration to reconcile humans’ damage to Earth’s freshwater. We also identify areas where research is needed.

## 1. Introduction

### 1.1. Worldwide Freshwater Quality Threats

Currently, worldwide freshwater health is increasingly threatened by unprecedented human, terrestrial, upland practices [1,2,3,4,5] and global climate change [6]. Drinking water and recreational resources are contaminated by emissions from non-point sources with various management practices [1,3,4,6]. Human settlements, industries and agriculture are the major sources of water pollution, contributing 54%, 8% and 38%, respectively [7]. This is especially concerning because water use is predicted to approach one-half of Earth’s capacity by mid-century [2] and any contamination may reduce the utility of these resources further. While many nutrients and pollutants are exported to water bodies through runoff, phosphorus (P), a limiting nutrient in freshwater ecosystems, is of particular concern because it is a non-renewable resource essential to crop production [8], which when excessively discharged from landscapes can have damaging effects on the ecology of freshwater lakes and streams. Soluble reactive phosphorus (SRP) stimulates the growth of algal and toxic cyanobacteria [9,10], causing eutrophication, which results in anoxic conditions [11,12,13], directly harming human and animal health [14]. While most of the solution lies in evolving upland practices, ecological engineering offers creative ways that recover and recycle phosphorus upland, supporting food security while mitigating eutrophication [15].

### 1.2. Relatively New Field of Myco-Phytoremediation

Though the role of fungi in ecosystem processes has long been recognized, mycoremediation is considered an emerging field. Bioremediation technologies, that originally harnessed bacteria to mitigate pollutants, have been a crucial tool in the last 60 years to filter contaminants from wastewater before discharge to surface water. Now, bioremediation involves a much wider group of organisms including fungi. Mycoremediation can serve as a mitigation approach for non-point source pollution that addresses the problem through source reduction, contamination event prevention, and pollutant interception upland of the receiving water body [16]. Research on mycoremediation has involved enhanced rhizosphere cycling and mineralization of heavy metals, pharmaceutical wastes, polycyclic hydrocarbons, agricultural wastes (pesticides and herbicides), phthalates, dyes, and detergents, when working in tandem with microbes [17]. Absent from this list is phosphorus, a ubiquitous agricultural pollutant of freshwater bodies. Given the role of P in water quality degradation, it is surprising that mycorrhizal fungi have not been used in repairing landscapes to facilitate P uptake from soil and thereby preventing it from loading to water bodies.

Phytoremediation, on the other hand, involves plants that remove various pollutants such as hydrocarbons, alkanes, phenols, polychlorinated solvents, pesticides, chloroacetamides, explosives, trace elements, toxic heavy metals, metalloids and landfill leachates [18,19]. Phytoremediation is a cost-effective and environmentally sound way to conserve soil and water resources as well as provide farmers with viable hay for their livestock [20] and other resources. Phytoremediation could be enhanced with appropriate arbuscular mycorrhizae fungi (AMF) [21] and ectomycorrhizae (ECM). Plant uptake can reduce P concentrations in soil solution and thus reduce the movement of dissolved P into surface waters.

When mycoremediation and phytoremediation are combined, a synergistic symbiosis is facilitated which also includes microbes [22,23]. In the literature, the reported utility is in remediating metals and PCBs [24,25,26,27]. To our knowledge, it has not yet been applied to P mitigation rigorously beyond pilot projects, hence case studies are few and far between.

### 1.3. Mycorrhizae

Mycorrhizae fungi are 400 million-year-old ecological engineers whose evolutionary success has been attributed to their ability to expand the rhizosphere of plants, enabling greater uptake of nutrients from surrounding soils [28]. Early research indicates mycorrhizal application in agricultural production reduces the amount of P fertility amendments required for plant growth, tantamount to source reduction. Influx of P in roots colonized by mycorrhizal fungi can be 3–5 times higher than in non-mycorrhizal roots [29]. Their effectiveness in agricultural landscapes, however, is variable given the wide variety of farm management systems and other factors that interfere with their success. Rillig et al. [30] advocates for the development of mycorrhizal technologies to enhance agroecosystems sustainably.

Mycorrhizal fungi are keystone mutualists in terrestrial ecosystems [31] whose ecological role in assisting recovery of severely disturbed ecosystems [18] is evident because they enhance P plant uptake in both crops and woody plants. Thus they could play an important role in myco-phytoremediation of phosphorus. This involves ecosystem engineering which harnesses nutrient exchange networks crucial to ecosystem succession and resilience [32]. This strategy, though still relatively novel in modern landscapes, has tremendous potential to be applied in the burgeoning field of reconciliation ecology [33], which acknowledges that, while ecosystems cannot be completely restored to their original state, they can be reestablished to reverse their degradation to return to a new balance [34].

Of the seven groups of mycorrhizae, the two most common in agricultural and forested lands [28] are also the most likely to be employed in myco-phytoremediation: AMF and ECM. While AMF and ECM provide similar services to the plant (i.e., improved access to P) [29], their hyphae differ in architecture and in how they transfer P to the plant [35]. In the AMF, the transfer is accomplished intercellularly and via intracellular arbuscules from extra-radical hyphae that extend directly into the soil beyond plant rhizosphere depletion zones [36]. In ECM, the transfer occurs via intercellular Hartig net hyphal networks surrounding epidermal and cortex cells while outside of the mantle, extra radical mycelia form extensive nutrient-absorbing networks in the soil [37,38]. It is well established that AMF and ECM greatly enhance the uptake of immobile soil nutrients such as P by plant root hosts [35,39,40] and improve soil properties. They also increase below- and above-ground biodiversity and provide pathogen resistance. This results in improved tree and shrub survival, better growth and establishment on moisture-, nutrient- and salt-stressed soils [41,42,43,44]. In addition, they facilitate plant succession [45,46]. Mycorrhizae growing around or in roots utilize carbohydrates from the host, and in return supply the host with P [29], water and other nutrients [47,48].

Additionally, when planting into AMF grasslands, tree and shrub species’ growth and survival is improved by inoculation with ECM specific to the species planned [49]. ECM presence can support native trees to endure aggressive non-native species’ presence [50] as well as play a critical role in the restoration of degraded sites [48]. Mycorrhizae can assist in decreasing P pollution in each component of the three-pronged strategy introduced above: source reduction via decreasing P amendment amounts needed, contamination event reduction by decreasing erosion through improved soil structure and vegetation establishment and pollutant interception via redirecting P into plant roots out of soil and water.

This paper provides an overview of current research on how mycorrhizae and their native hosts can mitigate water quality degradation. In researching the application of mycorrhizae to remediate phosphorus for water quality purposes, we found ample studies investigating mycorrhizal symbioses in crops such as sorghum, wheat, corn, clover [51] but few studies applying them specifically to address water quality issues. The scope of this paper is limited to P mitigation in agricultural and urban settings mainly within temperate climate regions. In particular, we present a survey of literature which highlights mycorrhizal services that would potentially be of utility in myco-phytoremediation of P in the context of best management practices for water quality improvement across landscapes. Different research fields use different terminologies for P species. We use SRP to mean the dissolved inorganic phosphorus pool, i.e., plant available orthophosphate. Inorganic phosphate includes this pool but also the adsorbed portion and precipitates of phosphate.

## 2. The Phosphorus Problem

Most P enters water bodies as non-point source pollution with overland flow and streambank erosion of legacy P [52] and from leaching of long-term barnyard manure-amended soils [53]. The urgency to address this is not only due to the increasing eutrophication of waters around the world but also due to the finite P resources that remain and the presence of abundant legacy phosphorus, accumulated in soils from past fertilizer and manure inputs. Legacy P resources could substitute manufactured fertilizers, preserve the finite phosphate rock reserves and gradually improve water quality [54]. Additional urgency is due to the fact that water quality improvement will be gradual as a result of the inherent lag time between the initiation of P mitigation and tangible water quality outcomes. These lag times can be attributed to the chronic and continual release of non-point source pollution (NPS) from soils enriched in P during past management [55,56]. For this reason, NPS watershed mitigation projects often fail to meet expected timetables for water quality improvement [57]. When managing for P mitigation, it is helpful to identify whether mitigation practices focus on total P (TP) or SRP. Technically, both are important: in deep lakes, bioavailable P is more threatening to water quality health, whereas the impact of particulate P is more significant in shallow waters, due to its resuspension ability. However, well-intentioned conservation measures that reduce particulate P (PP) losses are, they may unintentionally contribute to increases in ecologically damaging SRP loads [58]. This emphasizes the importance of paying attention to P speciation (organic P ranging from 35 to 70% [59]) in conservation practices. SRP is important to study separately from TP because this portion is immediately bioavailable in contrast to P associated with sediment or organic matter [60].

Typical sources of phosphorus are manure, fertilizer and compost, although P is also naturally present in soil minerals such as apatite [61]. Because manure and composts are often enriched in P relative to nitrogen and the stoichiometry of plant needs [62], P builds up in soils, which may lead to P saturation [63]. The phosphorus cycle is complex and there are soils that have vast reserves of total P that can exceed SRP 100-fold [64].

Hence a key challenge is how to raise the efficiency of agriculture to increase the availability of inorganic phosphorus (Pi) soil reserves to crop plants [65] while also reducing inputs. In agricultural soils, P use efficiency is low compared to the amount that is adsorbed to soil colloids where it is strongly held. Although P is rendered less mobile by sorption, it finds its way into water courses mainly by erosion of phosphorus-laden sediments [66].

A phosphorus source reduction approach involves meeting sufficiency recommendations based on soil tests [67,68]. Calculations of P removal as a function of crop, soil and management factors differentiate areas that may vary in P soil test levels (and resulting potential for P runoff). Doing so can inform large-scale applications using the P site index where P soil test levels cannot be determined for each specific tract of land [69].

Another strategy to reduce P fertilizer use, not often considered in soil fertility measurements, is to involve soil structure improvement that would increase organic matter storage and thus P storage which could become available to plants [70]. P sorption maxima have been correlated with carbon (C) from organic matter due to humic Fe, Al complexes responsible for increased P sorption [71].

Plants invest up to 20% of photosynthate in mycorrhizal symbioses [72] to obtain nutrients whose available forms are in short supply [28]. The mechanism by which mycorrhizae enhance nutrient uptake is through extending reach of plant roots via extensive hyphal networks, which can exceed distances of 11 cm from the host root [73], or by manipulating the chemical environment to release more phosphate from labile organic and inorganic sources [74,75].

## 3. Processes in the Phosphorus Cycle Where Mycorrhizae Affect P Availability

**Figure 1:**
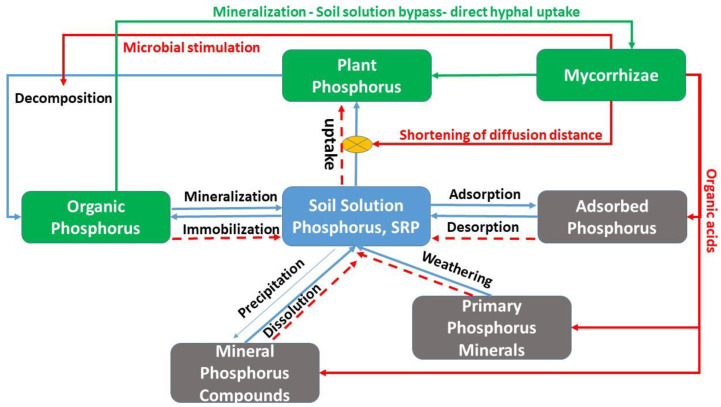
Influence of mycorrhizae on phosphorus cycling processes and pools. Red and green arrows are processes influenced by mycorrhizae. Broken lines show the net direction of reactions due to mycorrhizal effects.

Mycorrhizae participate in the main P cycling processes. A simplified version of the soil P cycle is depicted in Figure 1 and shows where mycorrhizae may influence the cycle. At the center of the cycle is orthophosphate in soil solution, also known as dissolved or soluble phosphorus or SRP. P in this pool comprises three bio-available species of the phosphate ion (H_2_PO_4_^−^, HPO_4_^2−^, and PO_4_^3−^). This pool is connected to all other compartments: vegetation, organic P, P sorption sites on Fe and Al oxides, and mineral compounds, so called secondary minerals, which form by precipitation of phosphate with Fe, Al, and Ca ions and release phosphate by dissolution. In addition, there is a phosphorus pool associated with primary P minerals (apatite) which releases P slowly and which may also be manipulated by ECM [76]. One could further split both the organic and the inorganic pools into two types of P: labile, fast-cycling and stable, slow-cycling P. The efficacy of mycoremediation via mycorrhizae may rely on catalyzing these pools to accelerate P extraction by plants which can subsequently be harvested to remove some P from the site. This form of mitigation is called myco-phytoremediation.

**Figure 2:**
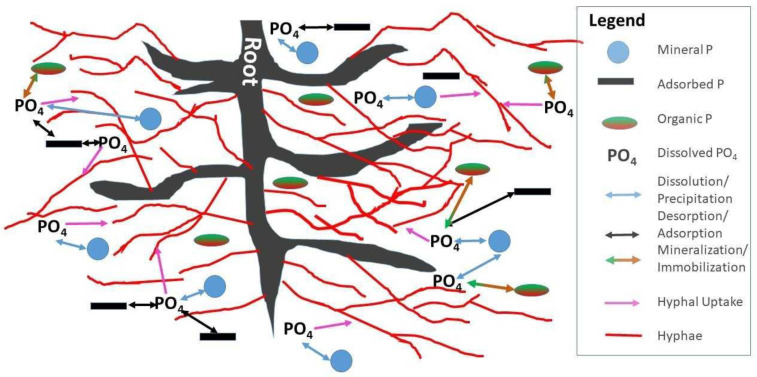
Interactions among spatially distributed organic, adsorbed, and particulate mineral phosphorus microsites, soil Scheme 4 and mycorrhizae hyphae.

Mycorrhizal fungi affect the P cycle by several mechanisms which can be understood as physical and biochemical. On the physical side, mycorrhizal hyphae increase the chance that dissolved phosphate is encountered by increasing diffusion of orthophosphate in solution into the root–hyphal network. There are several factors that contribute to this effect [77]: (i) AMF diameters are smaller than plant roots thereby increasing surface area to access a greater soil volume [73] than plant roots alone and reducing the diffusion distance; (ii) the constant turnover and new growth of AMF maximizes soil exploitation [78]; (iii) AMF with high affinities for P uptake, are highly efficient [79]; and (iv) once taken up by AMF hyphae, orthophosphate is converted into polyphosphate, which helps maintain a phosphate concentration gradient across the soil–hyphae boundary, assisting in P uptake [80]. Here it is helpful to consider the spatial distribution of P pools and their relationship to the distribution networks of roots and hyphae (Figure 2). On the one hand, the root–hyphae partnership has to compete for solution phosphate with microbial immobilization, sorption and precipitation. On the other hand, mineralization, desorption and dissolution locally liberate phosphate into soil solution; hyphae increase the chance that plants have agents in the place and at the time where and when these events occur (Figure 2).

Mycorrhizae-associated biochemical processes that increase plant uptake involve organic acids [81] that dissolve precipitates of phosphates and primary minerals [74] and phospholytic enzymes that help mineralize P from organic sources [82]. Recently it has been recognized that mycorrhizae may act in concert with other microorganisms in their mycorrhizosphere [76,77] to increase phosphate mineralization [83] similar to enhanced mineralization in the rhizosphere [81]. Biochemical processes can differ from the physical processes because they allow hyphae to take up phosphate directly from the organic residues, thus bypassing soil solution (green arrow in Figure 1). This may have important consequences for myco-phytoremediation (explained more below) as it releases plants from competition for P by adsorption and precipitation.

Erosion control is an effective way to prevent the movement of sediment-bound P into water bodies [84]. This is noteworthy since mycorrhizae affect soil structure on both micro and macroscopic levels. AMF produce glycoprotein glomalin, which binds soil particles into aggregates [85], remaining in the soil even after mycorrhizal death [86]. The increased aggregation reduces erosion by maintaining a porous yet stable soil structure [87]. Greater ECM activity can increase stable aggregate levels in the soil due to fungal hyphae growth [88] thereby enhancing soil restoration, driving plant community development [89], and hence can serve as a management tool to support restoration of boreal and temperate forest ecosystems [48] which includes buffers and vegetated drainageways.

**Figure 3:**
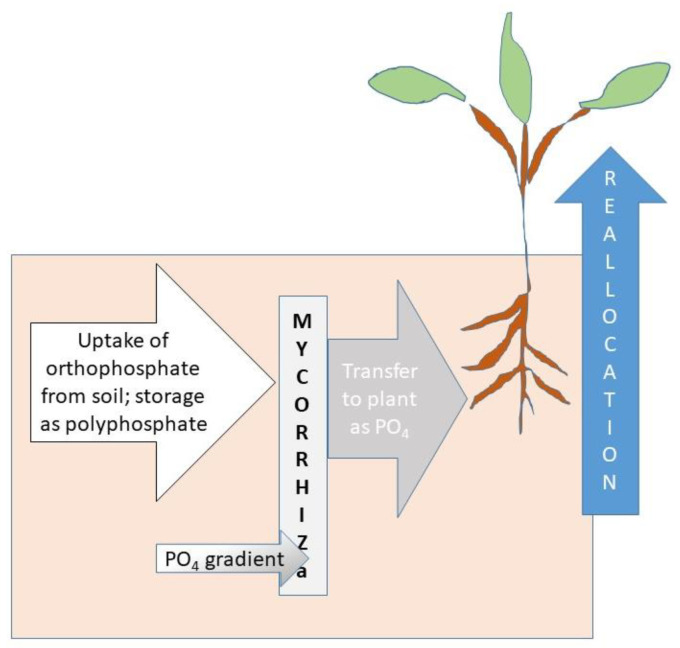
Multistep transfer of orthophosphate from soil through mycorrhizae to the plant.

A crucial task in P runoff mitigation is to accelerate P removal from where it has accumulated, over years of agricultural management, in crop fields, pastures, and buffers. This task can be aided by mycorrhizae through three steps: P uptake via mycorrhizae, P acquisition from the soil into storage, and P allocation to places in the plant where it is needed (Figure 3) [90,91]. Plant processes such as modifications in root structure, organic acid, proton, and phosphate production and activation of high affinity transporters affect P acquisition [92] as do mycorrhizae associations [93]. P utilization efficiency meanwhile is governed by P transport within the plant remobilization and internal P apportionment to maintain plant metabolism under low P concentrations [94,95]. It is important to note that these processes occur at spatially distributed microsites in the soil as shown in Figure 2.

Mycorrhizospheres and their composition significantly affect the mobilization of both inorganic particulate and organic P into the SRP pool. This depends on both the quality and the concentration of acids released by mycorrhizae [96]. Mycorrhizal fungi and roots also transport nutrients considerable distances [97].

The amount of SRP in the soil solution affects the efficacy of mycorrhizae to enter into symbiosis with the plant [98,99]. Increased SRP has inhibitory effects on development of external hyphae in soil core experiments [100] and thus the AMF are less likely to improve scavenging for P. In contrast when SRP is low, mycorrhizal infections and hyphal growth increase [101] resulting in greater plant P uptake and thus less chance of leaching of SRP [100].

In comparison to the sum of the other pools, soil solution phosphorus (SRP) can constitute as little as 0.1% of TP [64,102,103]. This is exacerbated by the fact that sorption rates of P are generally greater than plant uptake [104,105,106]. Thus newly applied phosphate becomes unavailable quickly, triggering the need for more P fertilization [107]. For this reason, agronomic assessments of plant available P have focused primarily on sorption-desorption and precipitation-dissolution [108]. The sorption-desorption reaction and the precipitation-dissolution reactions are equilibrium reactions. Thus, when the concentration of phosphate in soil solution is reduced by microbial immobilization and plant uptake, the two labile inorganic pools supply phosphate to maintain the partitioning ratio of solid phase to dissolved phase. In the presence of mycorrhizae, soil solution may then become a ‘pipeline’ for accelerated removal of P from the mineral pools to the plant.

Certain agricultural management practices such as avoiding overfertilization, and applying soil microorganisms which enhance P uptake like mycorrhizae fungi can move us toward more efficient P use [109]. Other strategies may rely on plants that utilize P more efficiently by selecting cultivars, plant breeding or genetic engineering [110].

The host plant’s P requirement and level of soil available P will also influence the extent of plant response to mycorrhizae [111]. AMF partners with 85% of plant families and can achieve a several-fold increase in plant uptake of phosphate compared to plants lacking these associations [36,83,112]. However, there is a wide spectrum of P uptake efficiency that can be attained by different AMF species [113,114]. Greater diversity of AMF is linked with ecosystem productivity and total P uptake potentially because different soil niches are occupied by different species [114].

Soil solution may not be the only source of P for AMF. The idea that this group of mycorrhizae might be saprotrophic [115] (i.e., they participate directly in the decomposition of organic matter to obtain carbon) is receiving renewed interest [116]. Mobilization of phosphate from organic matter may be a direct effect of the release of acid phosphatase [82]. However, other mechanisms have also been invoked. Mycorrhizae may prime or stimulate bacteria that live in the mycorrhizosphere by providing some of the photosynthate supplied by the plant [117]. Some species can also hydrolyze organic P compounds [118].

Increased plant uptake has been linked to reduction in phosphate leaching in several studies with AMF and thus has a direct effect on water quality. Zhang et al. [119] showed that SRP was reduced in both leachate and runoff by 11% and 81%, respectively. That study also found that losses of PP and dissolved organic P from rice mesocosms were much larger than SRP losses, but were also reduced. Bender et al. [120] found that AMF reduced leaching of SRP and unreactive P (total P minus SRP) by 31% over soils without AMF in grass mesocosms. Similar reductions with AMF were demonstrated by van der Heijden [121]. Martinez-Gracia [122] found that regardless of rainfall intensity mycorrhizae decreased P leaching losses by 50%. With climate change likely resulting in increased rainfall intensity in certain areas of the earth [123,124], mycorrhizae assist in resilient ecosystem response.

ECM is thought of as the group of mycorrhizae which can directly mineralize nutrients [115] from organic matter by releasing extracellular phospholytic enzymes [116,125]. Though they are not as ubiquitous as AMF, they partner with 10% of plant families, mainly woody species. However, ECM also increases P uptake from soil [74,126] likely protect water quality by conserving nutrients in forest ecosystems [115], such as riparian forested buffers.

Although mycorrhizae are strongly involved in phosphorus cycling, agricultural management affects mycorrhizal presence, abundance and effectiveness, influencing fertilizer need [127].

## 4. Mycorrhizae, Landscapes and Soils

Any design of a phosphorus mitigation strategy that involves mycorrhizae has to consider landscape position and soils which affect P availability and fate. In an ideal agricultural landscape, production fields are separated from water courses by a forested (or otherwise vegetated) riparian buffer [128], that attenuates the increased P in leachate when high fertilizer P is applied [129]. Each landscape element in the catena has a different role to play in P mitigation. Drainage class and vegetation need to be considered as variables for establishment of mycorrhizal communities. The mycorrhizal communities likely differ between high organic matter riparian forest including both AMF and ECM and the agricultural field of earlier succession dominated by AMF [130]. Drainage class per se may not affect mycorrhizal plant infections. In a study on soybean fields stretching across three soil drainage classes (poorly, somewhat poorly, and moderately well drained), more AMF spores were found in the more poorly drained than the better drained soils. But, there was no discernible difference in colonization of plant roots [131]. In agricultural systems where flooding diminishes vegetation, crops following the flood are P deficient early in the season. The lack of hosts during flooding may result in lower colonization rates by AMF [132]. Lack of vegetation during flooding is not likely to occur in forested riparian forests [133] and agricultural fields can be managed to provide hosts through rotations and cover crops [127].

However, drainage class may still enter into any myco-phytoremediation design because prolonged flooding in wetland riparian buffer, remobilizes P adsorbed to soil colloids. In particular, under anaerobic conditions ferric iron is reduced, releasing phosphate that would otherwise be strongly sorbed to feric oxides [134]. It is not clear whether mycorrhizae can help with recovering P released in this way.

In terms of the water mitigation paradigm, agricultural fields would be targets of source reduction as they are the primary recipients of P. However, in an area where agriculture was practiced for decades, it is likely the soil has sufficient P to be a source itself [135].

High SRP concentrations in agricultural fields are likely to reduce mycorrhizal infections [136]. Therefore, the amount of fertilizer P should be judicious [137,138,139]. Management of agricultural lands should consider the use of alternatives to inorganic P fertilizer to promote mycorrhizal growth and colonization [120,140].

Consequently, managing the field for mycorrhizae can reduce the amount of P fertilizer needed to achieve yield goals [127]. This includes reducing tilling and maintaining hosts by implementing crop rotation, and also choosing crops with root architecture efficient in accessing sufficient P and forming a symbiosis with AMF [101].

Oka [141] found that P application on soy beans could be reduced from 150 to 50 kg P ha^−1^ without yield loss when it followed wheat, an AMF mycorrhizal crop (*Triticum sativum*); then when followed by radish (*Raphanus sativus*), a non-mycorrhizal crop. The benefits may be due to better establishment of mycorrhizae–plant associations under the low soil P supply in the early season with increased uptake of P ensuing [142]. Application of excessive fertilizer at this time of the growing season may inhibit mycorrhizal infections [142] and should be avoided. Mycorrhizal cover crops may thus have several benefits to the plant. First, they provide hosts for mycorrhizae and a source of organic P, scavenged between cash crops. In addition, over time, the amount of sediment-bound phosphorus lost by erosion will diminish. Consequently, downslope P accumulations in riparian areas are minimized.

Although agriculture can be regarded as a myco-phytoremediation system for legacy P, agricultural practices affect mycorrhizae. The type and timing of tillage has been identified as one such factor. The role of fungi in plant nutrition and soil conservation is compromised when the formation and survival of propagules (i.e., spores, hyphae, colonized roots) are threatened though tillage, disrupting physical and biological properties of soil. Spores serve as “long- term” propagules when host plants are not present, whereas hyphae are the main source of inoculum when plants are present in undisturbed soil. Deep plowing can dilute propagules, reducing plant root inoculations, especially in autumn when hyphae are detached from the host plant. Conservation tillage can protect survivability and inoculation, thereby improving soil aggregation and P uptake [143].

The structure and texture of soils is also an important factor in whether AMF has significant impacts on leaching and erosion. In agriculture, it is important to look at the relationship between fertilization and runoff. AMF significantly reduced nutrient leaching after rainfall events in sandy grassland soils [121]. This research has important implications for soils with poor P sorption capacity such as sandy soils and other highly permeable soils or heavily manured soils [71], where P can be lost during rainfall events.

Furthermore, mycorrhizae can intercept P in soil solution before it leaves the root zone with deep percolation. In contrast to the many studies that assess the effect of mycorrhizae on plant uptake of P, only few of them report how mycorrhizae affect P leaching. This is usually not regarded as a major pathway of P export from a field because of the high affinity of phosphate [144] to soil surfaces. However, Asghari et al. [100] explained that sandy-textured soils are likely to provide little internal surfaces for P adsorption. In addition, soils that receive high P fertilizer may also leach phosphate [129]. Water quality in freshwater bodies is sensitive to even small amounts of P [145] and thus leaching may have a significant effect. Ashgari et al. [100] found that AMF can reduce leachate P from soil columns packed with a loamy sand. In another laboratory experiment Köhl and van der Heijden [144] found that the effect varied with AMF species probably due to differences in root colonization: the more root colonization the greater the growth of the plant and presumably the less P was leached. This is because AMF symbiosis assists plants with P uptake [140,146] through reaching beyond P depletion zones to access greater soil P reserves [74]. Plant response to mycorrhizal formation depends upon the extent of mycorrhizal development [47]. It is not clear whether the results of these controlled laboratory studies are directly transferable to processes that occur in the field where many other factors are in play; more research is needed here.

Mycorrhizae are involved in most aspects of P cycling as can be seen in Figure 1. Data from the literature that show the effect of mycorrhizae on plant uptake, leachate and soil concentration. For example, plant uptake can be enhanced by between 40 and several 100 s of percent, leachate P is reduced by up to 60% and extractable P by 15% in a growing season (Table 1). However, variations in both plant and mycorrhizae species greatly influence P removal from soil and leachate.

## 5. Riparian Buffers

It has long been recognized that a functioning riparian forest can retain nutrients exported from agriculture [128]. They have been proven effective in temporarily reducing agricultural P loads through settling sediments, microbial immobilization and plant uptake [147] and are associated with the recovery of impaired streams in agricultural watersheds [148].

However, riparian watersheds have been under strong development pressure. Conversion of these forests to cropland or grazing [149] has led to ecological impairment of these areas [150]. As a result the earth’s waterways are threatened by widespread loss of ecological services and functions and will require collective stewardship which involves ecosystem based solutions and technical strategies to improve water infrastructure [151]. Mycorrhizae have been proposed as technologies that could help with restoration [45]. A greenhouse microcosm experiment involving the grass *Phalaris aquatica L* investigated the effects of AMF on plant growth, nutrient depletion from soil and leaching via water. The results indicate that where P was added, P levels in both the soil and water were significantly lower in the mycorrhizal inoculated plants compared to the non-inoculated plants. These results suggest riparian management practices which promote mycorrhizae could help minimize nutrient loss. What is most significant about this study is that it occurs in Australia’s nutrient-challenged riparian ecosystems, demonstrating how increasing this below-ground diversity can support nutrient interception in areas which experience rapid influxes of nutrients [112]. In theory, mycorrhizae could access P released from labile pools in sediments from upland soils. ECM fungi, and AMF, can directly access organic phosphorus for the plant [116], thus bypassing soil solution where plants would face intense competition for P from sorption and microbial uptake.

Plant uptake in buffers and bioretention projects can be significant, depending on plant species, type, and age [152]. For example, P uptake in a riparian buffer by woody vegetation (*Populus deltoides* in this case) was higher than herbaceous vegetative uptake [152] and the P amount removed via harvest was 62 kg P ha^−1^ over four years; 63% higher than in a control stand of smooth brome (*Bromis inermis*). Willows are suggested frequently for phytoremediation projects [153] because they are fast growing and can endure wet sites. They also have increased transpiration rates [154], which make them good candidates for accumulating P in their biomass.

Storage of P in buffer strips is not forever and release of P occurs at different time scales. Release may be associated with seasonal cycles such as growing and senescence periods of vegetation and the associated decomposition of dead plant material, and release of phosphate from labile mineral pools during flooding events. Ultimately removal of P has to be managed by harvesting perennial vegetation [152,155], so called phytoextraction, to reduce or prevent remobilization of nutrients and the inevitable release of accumulated P [156,157,158]. Phytoextraction is the last step of phytoremediation that directly impacts water quality and provides economic incentives to the farmer [152,155].

Harvesting buffer zone grasses and woody biomass removes accumulated P and prevents P saturation, increasing P retention and decreasing SRP losses in surface runoff [159]. In particular, the shrub zone tends to be the most efficacious to harvest because woody vegetation has greater uptake potential than herbaceous vegetation [152]. The harvesting of plant biomass may further ensure greater species diversity in wet areas exposed to high levels of external nutrient loading [160]. Inoculation with AMF and ECM could increase plant uptake by several fold. Some plants lend 45themselves to harvesting better than others. Plant selection is important in all landscapes as it is in agricultural areas to remediate terrestrial pollution. The high P uptake efficiency of willows, makes them a prime candidate for coppicing, the cyclic removal of biomass from trees, because willows have been documented to uptake 33% more P when they host AMF [161].

## 6. Green Stormwater Infrastructure

In urban and suburban landscapes, green infrastructure systems require a phytoextraction element to combat the inevitable P saturation which occurs over time in buffers, constructed wetlands (CW), and bioretention systems [162]. Generally, only 20% of the world’s wastewater [163] is treated, with even less treatment occurring in low-income countries [164]. As urban areas grow, so do impermeable surfaces and hard piping systems, which increase peak flows, stormwater volumes, and pollutant loading to rivers and streams [156]. To alleviate pollution loads, many US cities have implemented best management practices (BMPs) that slow and treat runoff. Among these are measures ranging from green roofs to constructed wetlands (CW).

Green roofs provide a range of ecosystem services such as stormwater retention, temperature moderation, urban biodiversity, carbon sequestration, and enhanced aesthetics [157]. It is important that leachate from green roofs be filtered and monitored [165] Since P is almost universally found in higher concentrations (as much as 20 times) in their leachate than in conventional roof runoff [158]. Mycorrhizae can be effectively integrated into green roof design to help plants endure dry and nutrient poor conditions while providing erosion control, species diversity and nutrient mitigation [158].

Bioretention is a common BMP which involves stormwater flowing through a vegetated area with engineered soil mixes [166]. Bioretention cells help reduce peak flows and remove pollutants such as nutrients and metals, through physical filtration, sorption, plant uptake, and microbial reactions. A challenge with these has been that the bioretention soil mix can become a source of nutrients and thereby contribute to water degradation [167]. Mesocosm experiments found that ECM and AMF mycelium in bioretention media planted with *Carex stipata* reduced TP by 13–48% and SRP by 14–60% [168].

Like some riparian areas, constructed wetlands (CWs) are characterized by wet to inundated soils. Since the 1950s, CWs have been studied as low technology methods to treat wastewater from agriculture [169], residences [170], and industry. In domestic wastewater, these wetlands can be effective in removing P [13]. Encouraging studies that hint at the role of mycorrhizae in wetlands comes from rice paddy and CW research which shows that even in flooded conditions mycorrhizae participate in plant P uptake [171,172].

## 7. Summary of Research Results from the Literature

Table 1 shows the effect of mycorrhizae on a number of the P pools and cycling processes as reported in the literature cited above. There are several effects. First, mycorrhizal infections clearly cause an increase in plant biomass P [49,51,82,83,112,114,127]. However, in a companion greenhouse and field fungi exclusion experiment [49] where fungicide was applied to inhibit mycorrhizae, the results were not as clear cut. Two grass species, *Avena barbata* and *Stipa pulchra*, were used in this experiment. For *Avena barbata*, the shoot and root concentrations were diminished by the presence of mycorrhizae in the greenhouse, but not in the field experiment. Yet, the data showed consistently that for *Stipa pulchra*, P concentrations were greater in the mycorrhizal treatment regardless of the experimental setting. It is not clear whether these inconsistent results are artifacts of using a fungicide. However, the negative effects of mycorrhizae on plant P have also been reported by others for certain experimental conditions. These include large additions of P in sewage sludge [99] when additions exceeded 200 mg P/kg soil. Similarly, in an experiment with and without P additions, *Trifolium subterraneum* took in less P with mycorrhizae present when P was added [100]. This is in agreement with the concept that high concentrations of P may reduce mycorrhizal infection. Duration of experimental incubation also seemed to have been a factor in the response of P concentration in *Medicago trunculata*. At longer incubation periods, the effect of both root and shoot P were less after 49 than 35 days. The effect of mycorrhizal presence was negative for shoots after 49 days [113]. In another experiment, the effect of mycorrhizae was positive on total plant P (*Zea mays*) [128] throughout the growing season during a field study. Overall, however, mycorrhizae have positive effects on plant P uptake.

The effect of increased plant P uptake should translate into reduced soil P if no additional fertilizer is added. Because of the large amount of P stored, adsorbed to soil colloids, it is difficult to detect a decrease in the total P fraction in the soil. However, extractable P has been shown to be reduced when corn is inoculated with mycorrhizae and is grown with no P fertilizer. This is consistent with increased P uptake by the plants. Extractable soil P is not significantly different between mycorrhizal and non-mycorrhizal treatments when P fertilizer is added [101].

Consequently, losses of P from the soil as leaching or runoff would also be expected to be reduced when mycorrhizae are present. This has indeed been shown in several laboratory column studies [100,112,119,121,129]. Again, the amount of soil P differentiates the response of the plant–mycorrhizal association. In cases where P is more abundant, the effect of the mycorrhizae on leaching is less than when P concentrations are lower [100,121]. In one study, however, leaching losses of SRP increased or were the same when mycorrhizae were present [129]. In this same study, the pairing of plant species with mycorrhizal species also affected leaching. For example, in the combination of *Lolium multiflora* and mycorrhizae *Rhizoglomus irregular*, leaching increased by 45%, but for its combination with mycorrhizae *Funnelformis mosseae*, P leaching decreased by 19.5% [129]. However, when *Trifolium pratense* was combined with three mycorrhizae, no significant differences were observed [129]. Although P additions inhibited the effect of mycorrhizae on leachate P, additions of N did not. Finally, climate change induced increases in precipitation volume rendered the plant–fungi associations less effective in reducing P leaching, presumably because additional rainfall creates a greater chance for more P leaching [122].

## 8. Research Needs

Little research has been conducted on the deliberate incorporation of mycorrhizae into phytoremediation strategies for mitigating P loading to freshwater. In particular, research is needed into their role in restoring riparian buffers and subsequently in the interception of P by the mycorrhizae–plant communities. An important question in this context is “how do mycorrhizae influence the trajectory of succession” after the initial restoration plantings. Closely linked to this question is how much P can the plant community extract and whether removal of plant material is feasible while facilitating ecosystem recovery. Comparing restorations with high and low biodiversity may yield information on the efficacy of P mitigation in buffers with these additional practices. Succession may also be affected by the P status of the riparian area and thus the fate of any P accumulating plants [173] and their mycorrhizal association.

Another promising area in need of research involves the potential of source reduction to decrease fertilizer needs. Specific crop combinations, cover crops, and green manures can be used to reduce fertilizer needs. Some grain crops have the ability to mobilize P from unavailable pools and thus transfer P to subsequent crops as their residues decompose [165,174,175,176]. Some plants with efficient P uptake may be well suited for transfer or P from crop to crop [177]. These P hyperaccumulators crops include Indian mustard, alpine pennycress, alyssum, canola, tall fescue, poplar, annual rye grass, alfalfa and sunflower [18].

Unlike crop rotations, intercropping of P mobilizing and non-mobilizing plants [170,178] that hyperaccumulate P may enhance removal simultaneously. Mass balance studies where legumes, able to mobilize P, are intercropped with grains, that accumulate P, may identify crop combinations that reduce P losses from fields. Whether P accumulation by these plants is increased by mycorrhizae is not yet clear and merits further research. Recent studies report improved intercrop performance, especially legume-cereal mixtures, relative to monocrops, from enhanced P nutrition for one or more intercropped species. Research in crop sequences and intercrops enhancing P cycling and crop nutrition, considering crop-specific P acquisition mechanisms, microbial community action, soil property effects, amount of and form of P will help move this promising quiver of regenerative techniques forward for farmers to incorporate into their systems [77].

Although there seem to be some combinations of plants that can leverage the mycorrhizal associations for better P removal, there are examples of plants that suppress the establishment of the symbiosis. Studies have mainly focused on invasive plants that reduce AMF infections. For example, Himalayan impatience, *Impatiens glandulifera*, which has invaded both European and North American riparian areas interferes with mycorrhizae [179]. Similarly, *Reynoutria japonica*, a non-mycorrhizal plant suppresses mycorrhizae and reduces their diversity [180]. However, increases in mycorrhizal abundance and diversity have also been reported for some invasions [181]. A general statement on the effect of invasive plants on mycorrhizae cannot be made [182].

While there is debate about whether non-native species are ecosystem place holders during climate change or actually malaffect native habitats and threaten ecosystem resilience [183,184] certain exotic species such as *Phragmites australis* effectively uptake excess nutrients such as P. As a phytoaccumulator in areas of intensive vegetation [185] these species can be removed annually through harvest and then used as mulch to areas seeking more P input. Research involving this and native macrophytes which have been identified as excellent captors of P such as *Typha latifolia* [186] are worthy of further study.

One confounding factor in myco-phytoremediation that makes it difficult to compare results is that currently researchers use either commercial inoculant or inoculant extracted from the wild. There are distinctions in the effectiveness between and within these two sources of inoculant which is not yet clearly determined. Standardized studies that compare how commercial vs. locally gathered and propagated mycorrhizae affect P cycling may help interpret the results of these two experimental approaches.

## 9. Conclusions

As 400 million-year-old symbiotic weavers of ecosystems with now 80% of terrestrial plants, mycorrhizae hold the keys to reducing P pollution from upland accumulations. Researching specific plant–mycorrhizae associations for P removal from soils and applying these findings to critical source areas on farms, urban conduits, and suburban corridors can benefit water quality.

The mycorrhizal effects that have been quantified, such as plant uptake and reductions in soil and leachate concentrations, show promise for reducing phosphorus pollution by myco-phytoremediation. A holistic approach that combines source reduction, interception, and prevention should be considered across the landscape scale. This involves nutrient management based on precision farming, plant breeding, crop rotation, intercropping, microbial engineering, microbial–fungal–floral symbiosis, increased perennial green infrastructures, and deliberate harvesting. This integrated approach, known as ‘agro-engineering’ [54], facilitates reconciliation of anthropogenic disturbance while reestablishing above- and below-ground ecosystem services [187].

Mycorrhizal research in the context of water quality is scarce. Methods need to be developed and tested to help agriculture become more regenerative and urban stormwater infrastructure more effective. Tools are also needed which accurately assess current mycorrhizal presence in ecosystems to which land managers can respond accordingly. As we develop more understanding of what AMF and ECM taxa are present and how they react to different soil treatments, microbes and flora [109], a more informed use of mycorrhizae can be brought into terrestrial landscapes to mitigate phosphorus pollution.

## Figures and Tables

**Table 1:** The effect of mycorrhizae on plant uptake, leaching and soil P from studies carried out under different experimental conditions and with different objectives. Underscored show the physical quantity measured.

Study Context	Study Conditions	Phosphorus Quantity Measured	% Change with Mycorrhiza ^#^	Location	Ref. #
**Crop uptake**	Agro ecosystem *Triticum aestivum*, AMF	Phosphorus use efficiency	+85–102%	Uttar Pradesh, Haryana, India	[22]
**Growth of native grasses**	Field ecosystem and pots in greenhouse, *Stipa pulchra Avena barbata*, fungicide/no fungicide ^***^	Shoot P concentration [mg/g]		San Diego CA, USA	[49]
		**Field**			
		*S. pulchra*,	+22%		
		*A. barbata*	+68%		
		**Greenhouse**			
		Shoot P concentration			
		*S. pulchra*	+1.6%		
		*A. barbata*	−11.8%		
		Root concentration			
		*S. pulchra*	+24%		
		*A. barbata*	−15%		
**Mulch Experiment**	Pots, greenhouse *Trifolium repens Zea Mays* Fungicide/no fungicide ^***^	Plant P concentrations (%)		Morioka, Japan	[51]
		No Mulch	+28%		
		Living Mulch	+135%		
		Plant P (mg P/plant)			
		No mulch	+17%		
		Living mulch	+709%		
**Crop uptake**	Pots, AMF, *Allium fistolosum*	Plant P concentration [mg/g]	+194%	Haguromachi, Japan	[82]
		Plant uptake [mg P/pot]	+1525%		
**Effect of mycorrhizosphere bacteria on plant uptake**	Pots, corn (*Zea Mays*), AMF	P plant uptake [mg P/pot]		Denmark	[83]
		Shoots	+168%		
		Roots	+234%		
**Effect of sewage sludge P on plant uptake**	Pot, greenhouse *Glycine max* AMF	Shoot biomass P [mg/shoot]		Ohio, USA	[99]
		No P addition	+144%		
		150 mg P/kg addition	+125%		
		270 mg P/kg addition	−0.8%		
		420 mg P/kg addition	−16.9%		
**Effect of AMF on P leaching**	Packed columns, greenhouse, *Trifolium subterraneum* AMF	Leachate P [mg]		South Australia	[100]
		without added P	−60%		
		with added P.	0%		
		Plant P [mg]			
		without added P	+251%		
		with added P	−23%		
**Effect of mycorrhizae on crop uptake and extractable soil P**	Pot, greenhouse, corn *(Zea Mays*), AMF	Plant uptake (mg P/plant)		Quebec Canada	[101]
		**Hybrid**			
		P3979	+8.4%		
		LRS	+19.1%		
		LNS	+19.8%		
		Mehlich 3 extractable Soil P Concentration [mg/kg]			
		**Hybrids, no P fertilizer**			
		P3979	−5.1%		
		LRS	−14.4%		
		LNS	−10.5%		
		Mehlich 3 extractable Soil P Concentration [mg/kg],			
		**Hybrids, P fertilizer applied**	ns		
**Leaching mitigation**	Pots, greenhouses, *Phalaris aquatic*, AMF	Shoot P content (mg)	+150%	Southeastern Australia	[112]
		Root P content (mg)	+168%		
**Spatial differences in P uptake between AMF species**	Pots, *Medicago trunculata*, AMF	Plant P concentrations		Roskilde, Denmark	[113]
		*Glomus caledonium*			
		**Shoot**			
		35 days	+39%		
		49 days	−17%		
		**Roots**			
		35 days	+61%		
		49 days	+10%		
		*Scutetllospora calosporia*			
		**Shoot**			
		35 days	+39%		
		49 days	−12%		
		**Roots**			
		35 days	+84%		
		49 days	+40%		
**Differential effect of AMF species**	Pots, *Medicago tranculata*, AMF ^##^	P uptake [mg/plant]		Mallala, South Australia	[114]
		***Glomus mossae***			
		4 weeks	+1425%		
		8 weeks	+314%		
		***Glomus claroideum***			
		4 weeks	+625%		
		8 weeks	+193%		
		***Glomus intraradices***			
		4 weeks	+925%		
		8 weeks	+357%		
**P losses from field**	Microcosms *Orya sativa* L AMF	Leachate [kg P/ha] ^###^		Jiangsu, China	[119]
		Particulate P	−11.1%		
		Dissolved Organic P	−14.4%		
		SRP (PO_4_) ^*^	−81%		
		Runoff [kg P/ha]			
		Particulate P	−11.1%		
		Dissolved Organic P	−4.95%		
		SRP (PO_4_) ^*^	−11%		
**Nutrient cycling in presence of mycorrhizae**	Microcosms, Heath and Pasture communities, AMF	P in leachate [mg] ^###^		Switzerland	[120]
		**Pasture**			
		Added NH_4_	−14.2%		
		Added NO_3_	−38.5%		
		**Heath**			
		Added NH_4_	−68.4%		
		Added NO_3_	−63.4%		
**Leaching from grasslands**	Mesocosms, grassland, AMF	Reduction in leaching			[121]
		Low nutrient availability	~ 60%		
		High nutrient availability	ns		
**Climate Change Resilience**	Mesocosms, grassland communities, AMF	Leachate P [ug] ^###^		The Netherlands	[122]
		Moderate rain	−149%		
		High rain	−58%		
**Crop Uptake**	Pots, *Allium fistulosum* (Welsh Onion) AMF	Shoot concentration	+88%	Tozawa, Japan	[127]
**Crop uptake**	Agroecosystem Zea *Mays AMF*	Plant P [mg/plant] ^**^		Quebec, Canada	[128]
		**Year 1 Sample days**			
		22	+26.5%		
		48	+46.5%		
		72	+18.7		
		**Year 2 Sample days**			
		22	+19.4%		
		48	+14.2%		
		72	+41.8%		
**Nutrient Leaching**	Laboratory mesocosms. *Lolium multiflorum, Trifolium pratense*, sterilized soils AMF	Leachate Loss SRP [mg]		Zürich, Switzerland	[129]
		***Lolium multiflora***			
		*Claroideoglomus claroideum*	+14.2%		
		*Funnelformis mosseae*	−19.5%		
		*Rhizoglomus irregular*	+45.0%		
		***Trifolium pretense***			
		*Claroideoglomus claroideum*	ns		
		*Funnelformis mosseae*	ns		
		*Rhizoglomus irregular*	ns		
		Unreactive P			
		***Lolium multiflora***			
		*Claroideoglomus claroideum*	−10.8%		
		*Funnelformis mosseae*	+3.9%		
		*Rhizoglomus irregular*	ns		
		***Trifolium pratense***			
		*Claroideoglomus claroideum*	+29.9%		
		*Funnelformis mosseae*	+19.1%		
		*Rhizoglomus irregular*	+62.4%		
**Vegetative buffers**	Pot, *Salix, Populus* AMF	P stem content	+33%	Southern Quebec, Canada	[162]
**Bioretention**	Field mesocosms, *Carex stipata*, AMF/ECM commercial mix	Leachate mass rate (mg/hour) ^###^	−34%	Portland, Oregon, USA	[169]
**Crop uptake**	Microcosms, *Orya sativa* L. AMF	Plant P concentrations {mg/g] ^###^		Sweden	[171]
		**First growth stage**			
		Leaf	ns		
		Stem	+66%		
		**Continuous flooding**			
		No flooding	−19%		

ns = no significant difference; calculation of % change = (treatment − control)/control; ## also used leeks, but P uptake was 0, leaving the % change undefined; ### digitized from graphs using Image J (NIH, Bethesda, Maryland); ++ only the effect of AMF considered; * % difference represents an approximate estimate due to difficult digitization for PO_4_. Authors state that the differences were significantly different; ** data analyzed for unfertilized plots, fungicide treatment used as control; *** treatments consisted of fungicide (no to low mycorrhizal colonization) and no fungicide (high mycorrhizal colonization).

## Abbreviations

AMF	Arbuscular mycorrhizal fungi also known as Endomycorrhizae
BMP	Best management practices
CW	Constructed wetlands
ECM	Ectomycorrhizal fungi
NPS	Non-point source pollution
P	Phosphorus
Pi	Inorganic phosphorus
PP	Particulate phosphorus
SRP	Soluble reactive phosphorus, orthophosphate
TP	Total phosphorus
